# Music Lessons and Cognitive Abilities in Children: How Far Transfer Could Be Possible

**DOI:** 10.3389/fpsyg.2020.557807

**Published:** 2021-01-08

**Authors:** Franziska Degé

**Affiliations:** Department of Music, Max-Planck-Institute for Empirical Aesthetics, Frankfurt, Germany

**Keywords:** music lessons, music training, children, transfer, cognitive abilities

## Introduction

For many years there have been an interest in cognitive transfer effects and very controversial debates about its existence. Often, transfer is referred to as a special case of learning, but it is also absolutely possible to characterize transfer as something that is inextricably linked to learning and so happens regularly coupled with the learning process (for an overview see Klauer, 2010). Transfer is a learning or exercise effect that goes beyond the primary effect of practice. Its effects can be positive or negative. In learning and practicing processes both main and side effects can arise. Main effects can be observed in the learned domain and are summarized under the term trivial learning transfer (often also near transfer). Side effects affect domains that have been neither practiced nor learned, and these are called nontrivial learning transfer or far transfer. The main characteristic that differentiates between trivial and nontrivial transfer effects is their particular difference between the original learning situation and the transfer situation: a low difference for trivial transfer and a high difference for nontrivial transfer. The higher the distance or the range of the transfer, the greater its qualitative assessment and the less frequently it takes place. However, it is possible that nontrivial transfer occurs and Taatgen (2016) provides a very helpful framework to understand how this could happen. You can imagine the whole idea as a kind of overlap between two skills in small elements that are used to fulfill a task. People train specific cognitive skills and—as a by-product—they train a general cognitive skill as well. This so-trained general cognitive skill can be helpful for other specific skills. For example, a child learns a specific instrument (e.g., piano), and while practicing the finger movements that are important to play piano, which is a very specific skill, it also trains selective attention (a general skill that is also useful for other cognitive tasks). In Taatgen's theory, this is the case because of the small elements of information processing that can be reused. The basic parts of Taatgen's model are the so-called primitive information processing elements (PRIMs). These elements can be combined to build a PRIM rule and then PRIM rules can be compiled to include more PRIM rules and form a composite PRIM rule, and ultimately if all rules are put together, they build the rule for carrying out an operator. Since the PRIM rules refer to very small processing steps, they are independent of the overarching goal of a specific skill. In terms of transfer, this means that the operators for one task (e.g., the multicolumn addition) can be reused completely and others partially for another task (e.g., multicolumn multiplication).

With this approach it gets clearer that a complex pattern of information exchange can still be relatively independent of the specific skill or task and therefore also that specific skills that seem to be different can allow transfer. Moreover, it counts not only completely similar operators that can be shared but also partially shared ones. Therefore, also the transfer between skills that have no perfectly similar operators can be explained. Furthermore, this model gives us insight into what happens in early (only few repetitions of a task) vs. late transfer (many repetitions of a task). When you start learning a specific skill, transfer to another skill (i.e., early transfer) might be higher, because there might be a large overlap between operators (operators are not as task specific in early transfer). The longer you train a specific skill, the more you get proficient in things that concern task control and are highly specific for a particular skill. Hence, being an expert in something might limit the potential of transfer (late transfer) due to the high specificity of operators. This might be interesting in light of the paradox Schellenberg (2011) pointed out earlier in the discussion about transfer: Children taking music lessons enhance their intelligence and that musicians who make music for years are slightly better in some cognitive abilities than nonmusicians but not geniuses.

Additionally, information provided by Mähler and Stern (2006) about specific circumstances (use of mental tools, metacognitive control, analogies, and individual motivation) that might facilitate the occurrence of transfer effects supports the idea that far transfer (nontrivial transfer) might take place. Music lessons offer such specific circumstances (like training the use of mental tools or giving the opportunity to realize that deliberate practice is helpful for music learning and the next math test) that result in far-transfer effects. Above and beyond those facilitating circumstances, I argue that music lessons are special, because they are highly adaptive which might also promote transfer effects.

## Music Lessons and Cognitive Abilities

Indeed, several studies reported an effect of music lessons on cognitive abilities in children (Schellenberg, 2004; Moreno et al., 2011; Bugos and DeMarie, 2017; Frischen et al., 2019). These beneficial (side) effects of music lessons were small with respect to effect sizes, but long-lasting (Schellenberg, 2006b) and music specific, as control groups receiving sports training (Degé and Schwarzer, 2011), painting lessons (Moreno et al., 2009), or drama lessons (Schellenberg, 2004) did not show them.

It is far beyond the scope of this article to review all literature about music lessons, music training, musical abilities, and cognitive abilities. Hence, I will highlight three important controversial issues.

First, it seems striking that numerous areas of cognition appear to improve with music lessons and/or music aptitude (Schellenberg and Weiss, 2013). For some of these cognitive abilities, a connection to music lessons seems obvious (Schellenberg, 2011), like for listening skills (Corrigall and Trainor, 2009) or sensorimotor functions (Costa-Giomi, 2005). Other specific links, e.g., to language abilities (Patel, 2008), visual–spatial abilities (Rauscher and Zupan, 2000), mathematical abilities (Bahr and Christensen, 2000), or memory (Roden et al., 2014) are less obvious. The diversity of links raises doubts about their specificity. Schellenberg (2011), taking a generalist view, suggests that an association between music lessons and a domain-general cognitive ability might explain all reported specific associations. Indeed, there is evidence that music lessons are associated with domain-general cognitive abilities like IQ (Schellenberg, 2004, 2006b) and executive functions (Degé et al., 2011). Thus, it seems likely that fewer specific links than have been suggested actually exist. It might be possible that for example the less obvious links mentioned above are the ones that are explained by a domain-general ability rather than by a specific link.

Second, the available evidence for specific as well as domain-general connections between music lessons and cognition is mostly based on correlational or quasi-experimental studies that do not provide evidence for causation. Most studies therefore cannot state unequivocally whether it is music lessons that influence cognitive abilities or whether highly skilled individuals are more inclined to take music lessons (i.e., preexisting differences). Due to their readiness of mind, these high-functioning children perform well on all tests, while still having time to take music lessons, because their day-to-day school activities (e.g., homework) are less time-consuming. Such preexisting differences seem like an appealing and parsimonious explanation. So far, preexisting differences in variables like socioeconomic status (SES), IQ, or personality have been considered. For example, children who take music lessons have a higher SES than children who do not (Sergeant and Thatcher, 1974). However, most studies controlled for SES either by randomization or by statistical means, which calls this explanation into question. Concerning IQ, it has been suggested that highly intelligent children are more likely to take music lessons than other children (Schellenberg, 2011). However, children who have decided, but have not yet started, to learn an instrument did not differ in brain structure, cognitive abilities, motor abilities, or musical abilities from children who did not intend to take music lessons (Norton et al., 2005). Regarding personality, studies with children and adults revealed that amount of music lessons was significantly positively correlated with openness to experience, conscientiousness, and sometimes agreeableness (Corrigall et al., 2013; Corrigall and Schellenberg, 2015). As with SES, it is important for (future) studies to control for potential differences in personality. Some studies already did that and still found an association between music lessons and cognitive abilities (e.g., memory) (Degé and Schwarzer, 2017). Hence, preexisting differences cannot fully explain how music lessons promote cognitive abilities. Furthermore, they fail to explain the findings of a small number of convincing and comprehensive experimental studies that have reported small but significant effects of music lessons on IQ (Schellenberg, 2004), inhibition (Moreno et al., 2011; Bugos and DeMarie, 2017; Frischen et al., 2019), or phonological awareness (Degé and Schwarzer, 2011).

Third, this small impact is supported by some meta-analyses. Regarding spatial skills, meta-analyses found an effect of music listening and music lessons (Hetland, 2000a,b). Other meta-analyses revealed either a strong correlation between music training and reading skills, but no reliable results for experimental studies (Butzlaff, 2000), or a modest but significant positive effect of music training on reading skills (Standley, 2008). However, when reading related outcome measures were investigated separately, a small impact of music training on phonological awareness (but not reading fluency) could be demonstrated (Gordon et al., 2015). Two more studies that investigated effects of music lessons on a number of cognitive domains rather than isolated aspects yielded conflicting results. While Benz et al. (2015) reported small enhancing effects on various cognitive abilities, Sala and Gobet (2017) found no influence of music lessons on cognitive abilities at all. What might be critical about the conclusion of Sala and Gobet (2017) is their viewpoint on all cognitive aspects as one. If you have a closer look at single cognitive abilities, the effect sizes are higher than for an overall measure. This might speak in favor of more solid transfer effects between music lessons and particular cognitive abilities. Moreover, small effect sizes for nontrivial transfer effects seem relatively plausible, because of their higher transfer distance. However, they still might be important. Additionally, they report that studies in this field of research could be done by more proficiently adopting a more sophisticated methodology. Although I would agree in general that for a lot of studies there is room to develop, I am wondering how far it is reasonable to use those studies to draw firm conclusions about far-transfer effects of music lessons on cognitive abilities. I think it would be rather plausible to conclude that it needs better studies to base future meta-analysis on, instead of concluding there is no effect at all.

All in all, I would summarize the field of music research by assuming that there are some small but interesting transfer effects of music lessons and cognitive abilities and that it is important to understand how they work in order to broaden our knowledge about them.

There are some frameworks or theoretical approaches that might help to understand transfer effects of music training. I will briefly mention two of them (OPERA hypothesis and multimodal integration) before discussing an explanation that seems for me very seminal to understand transfer effects of music lessons in children in a different way. The OPERA hypothesis by Patel (2011) was originally developed for the link between music and language. Patel (2011) has formulated a kind of framework that generally explains the different factors that are involved in the emergence of relationships between music and language. The five OPERA factors Overlap, Precision, Emotion, Repetition, and Attention are features of music or music making that facilitate the occurrence of transfer effects of music to language. However, this music-and-language framework might partly also hold true for transfer in general. Additionally, in a more neurological approach the idea of multimodal integration due to the multisensory activation of making music is put forward. Music making relies on multiple sensory modalities and the simultaneous integration of multisensory information (e.g., auditory, visual, and somatosensory) is needed to monitor progress and success. Hence, music practice is associated with structural and functional changes in the brain (Jäncke, 2009a). These changes can occur because neural plasticity leads to use-dependent regional growth and structural adaptation in response to intense environmental demands. Gaser and Schlaug (2003), for example, reported differences in gray matter distributions in musicians, amateur musicians, and nonmusicians in motor regions, auditory regions, and visual regions. The activation of many systems in parallel, on the one hand, results in brain changes and, on the other hand, might foster more general learning transfer (Green and Bavelier, 2008).

Both approaches consider the complex demands of music making. However, I would like to introduce an approach that emphasizes a slightly different point.

Among other explanations of potential transfer effects of music training, there is an interesting approach that might help to understand the impact of music lessons on cognitive abilities in children: the *qualitatively different experience explanation* (Schellenberg, 2006a). It is assumed that children who take music lessons make qualitatively different experiences compared to children not taking music lessons. Music lessons offer specific experiences (e.g., reading musical notation, doing auditory discrimination, memorizing musical notation and auditory passages, training of fine-motor skills, and gaining knowledge about musical structure), which train specific skills (e.g., executive functions). Regular practice of all these skills in childhood may have a positive effect on cognitive development. In short, cognitive benefits may stem from these “musically trained” abilities. What questions this postulation is the fact that some of these trained abilities are not specific to music (e.g., training of fine-motor skills); therefore other nonmusical extracurricular activities training such abilities should yield similar effects on cognition. According to Schellenberg (2004, 2006b), this was not the case. These findings point toward something special about music lessons that other nonmusical extracurricular activities cannot provide, but as yet, it is unclear what this is.

What I would like to stress a bit further are the implications of the qualitatively different experience hypothesis. It postulates that a limited set of abilities is trained by music lessons. I would argue that the set of skills subsumed under executive functions strongly overlaps with the set of abilities trained in music lessons. Music making requires executive functions (Jäncke, 2009b) and seems to have the potential to train them (Bugos et al., 2007; Bialystok and DePape, 2009; Degé et al., 2011; Moreno et al., 2011; Roden et al., 2014; Slevc et al., 2016; Jaschke et al., 2018). This idea is strikingly appealing, because it would represent a parsimonious explanation for the general and specific links between music lessons and cognition. Schellenberg and Peretz (2008) already postulated that executive functions could explain the link between music lessons and IQ, because music lessons train executive functions, and these are involved in nearly all tasks (Hannon and Trainor, 2007). This mediating hypothesis was tested and is possibly true (Degé et al., 2011), but see Schellenberg (2011). In this respect I would even go further and postulate that most of the potential associations between music lessons and cognition in children regardless of being general (i.e., IQ) or specific could be explained by executive functions (Degé et al., 2017). Furthermore, this idea is helpful in speculating what could be so special about music.

## Discussion: Adaptability

Getting back to the issue of whether music is special in any regard, I would not argue that other nonmusical extracurricular activities cannot improve cognition (e.g., tennis) (Ishihara et al., 2017) but that music lessons may be more efficient. The important difference between other activities and music lessons might be their remarkable adaptability, i.e., the fit of difficulty level between a musical piece and a students' ability: Music is normally taught in one-on-one settings or small groups, and teachers attempt to adapt their teaching to the ability of the music student(s). Moreover, assignments for daily practice are typically doable and designed to become incrementally harder in adaptation to the student's progress. Importantly, research not focusing on music (Klingberg et al., 2002) has suggested that successful enhancement of an ability, in their case executive functions, depends on the adaptability of the applied training. Hence, the specific “something” about music lessons might be their extremely adaptive nature. Other leisure time activities might not be as adaptive due to larger groups (e.g., soccer), not as flexible assignments (e.g., drama lessons with a given date for a performance), and fit of difficulty level (arts lessons in which it takes time until the feedback of mastery, i.e., the picture, is finished). As already mentioned, for successful music making, executive functions are important (Jäncke, 2009b). They are needed for monitoring progress, practicing music in an organized and disciplined way, inhibiting competing motor impulses, and switching to new rules when accidentals are inserted, or switching from playing one instrument to another. Hence, in music lessons, executive functions are trained in an adaptive way as well, which may be a particularly effective way of training according to Klingberg et al. (2002).

Thinking about it in a broader sense, I would put forward that it is the highly adaptive nature of music making that gives rise to general transfer/far transfer. This adaptability ensures that the student is more or less constantly trained in the zone of proximal development, the best *window* for successful learning: This zone is defined as the distance between a child's current developmental level as determined by independent problem solving and the next higher level as determined by problem solving under adult guidance or in collaboration with more capable peers (Wygotski, 1978, p. 86). In the Vygotskian framework, successful learning is slightly ahead of development (adapted to an individual's abilities) and takes place most effectively in the zone of proximal development.

I would like to highlight the factors that make it so likely for musical learning to consistently take place in this developmentally desirable zone. First, new milestones are often reached during music lessons: A good music teacher fits the to-be-learned materials to the learner's current skill level in a way that something new is mastered with the help of the music teacher within the lessons (i.e., a milestone). While for learning this milestone the teacher is needed, the mastery of it will (hopefully) take place at home during independent practice of the new skills. This training routine, when mediated by small group sizes and good teachers, ensures learning in the zone of proximal development. Second, what helps to keep music making in the zone of proximal development is its special form of feedback. Student and teacher receive immediate auditory feedback about their skills. If you like it or not, you hear immediately whether you have mastered a new piece or not. Third, the immediate feedback gives the opportunity to work on an issue directly as it comes up.

Taken together, the adaptability and the immediate feedback that allow for training in the zone of proximal development might be what makes music lessons special. This might be what enables them to affect cognition (e.g., executive functions) in a far-transfer kind of fashion.

It is highly debated if general transfer or general learning exists, and if yes, under what conditions. Green and Bavelier (2008) pointed out that general learning can take place if the learning paradigm is complex and “real world” enough, like video game training (needs to be games that are complex and not predictive, engaging, and difficult enough; so not every game would have this effect), athletic training, or musical training. They proposed that in addition, task difficulty, motivation and arousal, feedback, and variability are important contributors to general learning. I would in general agree to their ideas but argue that adaptability and its consequences might be the most important factor for enhancing effects of training. Adaptability can be directly related to their conditions: It can keep a task complex and difficult (always training in the zone of proximal development), it can produce motivated and aroused learners (oriented on the mastery of the next step), and it is based on feedback as well as variability. Video game training and sports training share an adaptive nature with music training to a certain extent, video gaming probably even more, but music training is more or less unique in its high amount of adaptability.

## Author Contributions

The content of the paper was developed by FD. The manuscript was written by FD.

## Conflict of Interest

The author declares that the research was conducted in the absence of any commercial or financial relationships that could be construed as a potential conflict of interest.
